# Gene Expression Correlates with Process Rates Quantified for Sulfate- and Fe(III)-Reducing Bacteria in U(VI)-Contaminated Sediments

**DOI:** 10.3389/fmicb.2012.00280

**Published:** 2012-08-09

**Authors:** Denise M. Akob, Sang Hyon Lee, Mili Sheth, Kirsten Küsel, David B. Watson, Anthony V. Palumbo, Joel E. Kostka, Kuk-Jeong Chin

**Affiliations:** ^1^Florida State UniversityTallahassee, FL, USA; ^2^Department of Biology, Georgia State UniversityAtlanta, GA, USA; ^3^Institute of Ecology, Friedrich Schiller University JenaJena, Germany; ^4^Oak Ridge National LaboratoryOak Ridge, TN, USA

**Keywords:** transcript level, uranium reduction, active sulfate-reducing bacteria, active metal-reducing bacteria

## Abstract

Though iron- and sulfate-reducing bacteria are well known for mediating uranium(VI) reduction in contaminated subsurface environments, quantifying the *in situ* activity of the microbial groups responsible remains a challenge. The objective of this study was to demonstrate the use of quantitative molecular tools that target mRNA transcripts of key genes related to Fe(III) and sulfate reduction pathways in order to monitor these processes during *in situ* U(VI) remediation in the subsurface. Expression of the *Geobacteraceae*-specific citrate synthase gene (*gltA*) and the dissimilatory (bi)sulfite reductase gene (*dsrA*), were correlated with the activity of iron- or sulfate-reducing microorganisms, respectively, under stimulated bioremediation conditions in microcosms of sediments sampled from the U.S. Department of Energy’s Oak Ridge Integrated Field Research Challenge (OR-IFRC) site at Oak Ridge, TN, USA. In addition, *Geobacteraceae*-specific *gltA* and *dsrA* transcript levels were determined in parallel with the predominant electron acceptors present in moderately and highly contaminated subsurface sediments from the OR-IFRC. Phylogenetic analysis of the cDNA generated from *dsrA* mRNA, sulfate-reducing bacteria-specific 16S rRNA, and *gltA* mRNA identified activity of specific microbial groups. Active sulfate reducers were members of the *Desulfovibrio*, *Desulfobacterium*, and *Desulfotomaculum* genera. Members of the subsurface *Geobacter* clade, closely related to uranium-reducing *Geobacter uraniireducens* and *Geobacter daltonii*, were the metabolically active iron-reducers in biostimulated microcosms and *in situ* core samples. Direct correlation of transcripts and process rates demonstrated evidence of competition between the functional guilds in subsurface sediments. We further showed that active populations of Fe(III)-reducing bacteria and sulfate-reducing bacteria are present in OR-IFRC sediments and are good potential targets for *in situ* bioremediation.

## Introduction

Mining and milling of uranium for nuclear weapons production has resulted in widespread uranium contamination in subsurface environments across North America, South America, and Eastern Europe (Abdelouas et al., 1998). Oxidized uranium, U(VI), is highly soluble and toxic, and a potential contaminant to local drinking water supplies (Palmisano and Hazen, 2003). Immobilization of oxidized uranium can be achieved in contaminated groundwater through the reduction of U(VI) to insoluble U(IV) by indirect (abiotic) and direct (enzymatic) processes catalyzed by microorganisms (Wall and Krumholz, 2006; Kostka and Green, 2011). Current remediation practices for dealing with uranium contamination aim to promote U(VI) immobilization via natural attenuation or the biostimulation of indigenous microorganisms through a combination of pH neutralization and/or the addition of electron donor (Finneran et al., 2002; Anderson et al., 2003; Wilkins et al., 2006; Groudev et al., 2010).

Dissimilatory Fe(III)-reducing bacteria (FeRB) and sulfate-reducing bacteria (SRB) comprise two major groups which are capable of U(VI) reduction (Tebo and Obraztsova, 1998; Lovley et al., 2004; Sani, 2004; DiChristina, 2005b; Payne and DiChristina, 2006; Wall and Krumholz, 2006). Both FeRB and SRB can directly reduce U(VI) by using it as an electron acceptor, and a subset of these groups have been shown to conserve energy for growth via U(VI) reduction (Lovley et al., 2004). In addition, the products of microbial Fe(III) and sulfate reduction, Fe(II) and hydrogen sulfide, can react abiotically to reduce U(VI) (Liger et al., 1999; Hua et al., 2006). Therefore, FeRB and SRB are considered to have a high bioremediation potential in U(VI) contaminated subsurface sediments. Although prior research has linked the activity of these functional guilds to U(VI) immobilization in contaminated subsurface environments (Anderson et al., 2003; Wu et al., 2006; Akob et al., 2008; Cardenas et al., 2010), it remains difficult to directly relate the *in situ* activity of specific microbial groups to the environmental controls of the processes. In order to exploit the activity of FeRB and SRB for bioremediation, there remains a need to develop quantitative tools for monitoring the metabolic activity of these microbial groups in subsurface environments. Quantifying the *in situ* activity of Fe(III) reducers is particularly problematic and a molecular proxy for Fe(III) reduction has not yet been verified or calibrated with biogeochemical rate measurements in any sedimentary environment.

A promising strategy for quantifying the *in situ* metabolic activity of SRB and FeRB would be to monitor mRNA transcript levels of key genes involved in sulfate or Fe(III) reduction. The dissimilatory (bi)sulfite reductase (*dsrAB*) gene is highly conserved among sulfate-reducing prokaryotes (*Bacteria* and *Archaea*) and codes for the dissimilatory (bi)sulfite reductase, which is responsible for the rate-limiting step of sulfate reduction (Wagner et al., 1998). Levels of mRNA for *dsrAB* genes were shown to increase in pure culture studies of dissimilatory SRB as rates of sulfate reduction increased (Neretin et al., 2003; Villanueva et al., 2008) and correlated with the activity of SRB in petroleum-contaminated marine harbor sediments (Chin et al., 2008). In the case of Fe(III) reduction, no single respiration pathway has been identified as FeRB can reduce insoluble Fe(III) oxides via direct enzymatic reduction, electron shuttling pathways, or by solubilizing metals with organic ligands (DiChristina, 2005a). One approach is to target and monitor functional genes of important groups of FeRB known to be involved in Fe(III) reduction and to be abundant in contaminated subsurface sediments. Members of the Geobacteraceae family are one such group and cytochromes which are involved in Fe(III) reduction have been identified in pure cultures of different *Geobacter* species. However, comparative analysis of available *Geobacteraceae* genome sequences has revealed that these cytochromes are not conserved throughout the Geobacteraceae family (Butler et al., 2010). Furthermore, it has been known that an outer-membrane cytochrome o*mcB* expression patterns were largely affected by environmental fluctuations, such as changes in electron acceptor availability, suggesting that monitoring *omcB* transcripts in *Geobacter*-dominated environments would not provide an accurate indication of rates of Fe(III) reduction (Chin et al., 2004). In addition to these characteristics, the *Geobacteraceae* family does contain a phylogenetically distinct functional gene, the citrate synthase (*gltA*) gene, that codes for an enzyme involved in the incorporation of acetate into the tricarboxylic acid (TCA) cycle (Bond et al., 2005; Holmes et al., 2005). The *Geobacteraceae*-specific *gltA* gene is a good target for this group of FeRB because it is more similar to eukaryotic citrate synthase genes (Methe et al., 2003; Bond et al., 2005) distinguishing it from other prokaryotic FeRB and heterotrophs. Measurements of *gltA* transcripts were used as a proxy for the activity of Geobacteraceae during bioremediation of uranium-contaminated groundwater (Holmes et al., 2005) and sediments (Akob et al., 2008). However, no study of metal or sulfate reduction in subsurface sediments has directly linked transcript level with process rates determined by geochemical methods. Therefore, in this study we quantified the transcript level of functional genes as a molecular proxy for the metabolic activity of Geobacteraceae-related FeRB and SRB in parallel with determining process rates and the abundance of predominant electron acceptors in field samples.

## Materials and Methods

### Site and sediment sample description

The study was conducted at the Oak Ridge Integrated Field Research Challenge (OR-IFRC) site of the U.S. Department of Energy’s (DOE) Subsurface Biogeochemistry Research program, which is located adjacent to the Y-12 industrial complex within the Oak Ridge National Laboratory (ORNL) reservation in Oak Ridge, Tennessee. For a detailed site description refer to the OR-IFRC webpage (http://www.esd.ornl.gov/orifrc/). Sediments were sampled from borehole FB107, within the Area 2 experimental plot, 5–7 m below the surface, on September 12, 2007 and from borehole FB124, Area 3 experimental plot, 1.23–15.08 m below the surface, on February 7, 2008 within the saturated zone, using a Geoprobe equipped with polyurethane sleeves lining the corer. Cores were aseptically sectioned under strictly anoxic conditions in an anaerobic chamber (Coy Laboratory Products, Grass Lake, MI, USA) and stored anaerobically in gas-tight containers at 4°C prior to overnight shipment to Florida State University. Sediment core sections were subsampled for total nucleic acid extraction and chemical analysis in a Coy anaerobic chamber immediately upon arrival at Florida State University.

### Microcosm design and sampling

A microcosm experiment was performed with Area 2 FB107 sediments to assess the activity and composition of microbial communities under simulated bioremediation conditions. Microcosms were prepared as previously described (Akob et al., 2008) except that the sediment was diluted 1:5 (w/v) with sterile, anaerobic artificial groundwater (composition per liter: 1.0 g NaCl, 0.4 g MgCl_2_·6H_2_O, 0.1 g CaCl_2_·2H_2_O, 0.5 g KCl, 1 ml selenite-tungstate solution, 1 ml trace elements solution, and 5.0 mM NaHCO_3_; modified from Widdel and Bak, 1992). Three replicate microcosms were established for each of the following treatments: amendment with ethanol, amendment with ethanol + molybdate, amendment with acetate, and amendment with acetate + molybdate; with molybdate added to inhibit sulfate reduction. Sulfate (2.5 mM final concentration), molybdate (5 mM final concentration), and electron donors (5 mM ethanol or acetate) were added from sterile anaerobic stocks.

Microcosms were sampled by removing ∼5 ml every 1–3 days using a sterile syringe equipped with an 18G needle under a H_2_/N_2_ atmosphere in a Coy anaerobic chamber. HCl extractable Fe(II) content was measured as previously described (Akob et al., 2008). Porewaters were extracted for determination of uranium and sulfate concentrations and carbon substrate utilization as previously described (Akob et al., 2008). At select time points, samples from each replicate microcosm were collected and pooled for cultivation-independent microbial community characterization by centrifuging samples at 7,000 × *g* for 7 min, discarding the supernatant, and freezing the solid phase at −80°C immediately until nucleic acid extraction.

### Chemical characterization of sediments

Soluble chemistry was determined on sediment samples from each depth interval by extracting sediment in a 1:1 (w/v) ratio in deionized water followed by a 1 h incubation shaking at 200 rpm. Samples were centrifuged at 5,000 × *g* for 5 min, followed by filtration through a 0.22-μm nylon syringe filter. The liquid phase was then analyzed for ${\text{SO}}_{\text{4}}^{\text{2}-}$ by the BaSO_4_ turbidimetric method (Rodier, 1975), for nitrate using the colorimetric method described by Cataldo et al. (1975), and the pH was measured with a calibrated digital pH meter (McLean, 1982). Samples for uranium determination were acidified with nitric acid and stored at −20°C prior to kinetic phosphorescence analysis using a KPA-11 analyzer (Chem-Chek Instruments, Richland, WA, USA; Brina and Miller, 1992).

Total iron and Fe(II) in each depth interval were determined by oxalate extraction and colorimetric quantification using ferrozine, as previously described (Kostka and Luther, 1994). In brief, sediment samples for Fe(II) determination were kept under strictly anaerobic conditions and were extracted in anaerobic oxalate (0.2 M ammonium oxalate, 0.2 M oxalic acid, pH 2.5) for 4 h in the dark, shaking at 200 rpm. After extraction, samples were centrifuged for 5 min at 5,000 rpm and the extract was added to ferrozine reagent (50 mM HEPES, 0.1% ferrozine, pH 7.0). The extract and ferrozine were incubated for 10 min in the dark then measured spectrophotometrically at 562 nm. Sediment samples for total Fe determinations were dried aerobically then extracted in oxalate in the dark at room temperature by shaking at 200 rpm for 4 h. After extraction, samples were centrifuged for 5 min at 5,000 rpm and the extract was reacted with total Fe reagent (1% hydroxylamine hydrochloride in ferrozine) for 4 h in the dark prior to measurement on a spectrophotometer at 562 nm (Green et al., 2010).

### Extraction of total RNA, mRNA, and DNA

Total RNA was extracted from a total of 3 ml of microcosm sediments and 3 g of *in situ* borehole sediments, and the recovered RNA was treated with RNase-free DNase to remove any contaminating DNA according to previously described methods (Chin et al., 2008). In order to prevent coextraction of inhibitory compounds such as humic acids and clay minerals polyvinylpyrrolidone K25 (PVP) was used during the total RNA extraction. Then, the extracted RNA was purified with a Sephadex (G-100) column filtration. To further purify the RNA, it was precipitated with sodium acetate, glycogen, and ethanol at −80°C for 30 min, purified RNA was recovered by centrifugation. The mRNA was enriched and isolated by a magnetic bead hybridization method with MICROB Express purification system (Applied Biosystems, Foster City, CA, USA), and large ribosomal RNA (16S and 23S rRNA) were removed by this protocol procedure. mRNA isolation using magnetic bead hybridization procedure followed additional purification provided high purity of mRNA. To remove any residue of small RNAs (including tRNA and 5S rRNA), the enriched mRNA was further purified with the glass fiber-based filtration method with the MEGAclear purification system (Applied Biosystems). DNA contamination was checked with agarose gel electrophoresis following reverse transcription-polymerase chain reaction (RT-PCR) by performing control experiments in which no reverse transcriptase was added to the extracted RNA before the PCR step. Concentration of RNA and mRNA was determined and the purity was checked with a Biophotometer (Eppendorf, Hamburg, Germany) and a NanoDrop 2000 Spectrophotometer (Thermo Scientific, Wilmington, DE, USA). Purified RNA and mRNA were stored at −80°C. Total DNA was extracted from a total of 2 ml of microcosm sediments according to previously described methods (Chin et al., 2008).

### PCR primers

The primers used in this study are listed in Table 1. The primer pairs CS375nF/CS598nR and DSR1F/DSRQP3R were used for real-time PCR quantification of *Geobacteraceae*-specific *gltA*, and bacterial and archaeal *dsrA* mRNA transcripts, respectively. The primer pairs CS18nF/CS821nR and CS1Fdeg/CS2Rdeg were used for reverse transcription PCR of Geobacteraceae-specific *gltA* transcript and for phylogenetic analysis. The primer pair DSR2MF/DSR4R was used for PCR amplification of bacterial and archaeal *dsrAB* DNA and for phylogenetic analysis. The primer pair DSV230f/DSV838r was used for reverse transcription PCR of sulfate-reducing bacteria-specific 16S rRNA and for phylogenetic analysis. Primers were evaluated for quantitative real-time PCR as described earlier (Chin et al., 2004). The resulting conditions were experimentally checked using genomic DNA isolated from *Desulfovibrio desulfuricans* subsp. *desulfuricans* and *Geobacter metallireducens* cultures for *dsr* gene and *gltA* gene, respectively. All primers were synthesized by Integrated DNA Technologies (IDT; San Jose, CA, USA). To determine whether the primers were suitable, gene-specific qualitative PCR was performed before quantitative PCR. PCR products were amplified from cDNA generated by reverse transcription with the appropriate primers using the following conditions: 95°C (5 min); 40 cycles of 95°C (40 s); 52°C (1 min); 72°C (1 min) followed by a final extension at 72°C for 10 min. Cloning and sequencing verified the specificity of PCR products. Only the primer combinations, which amplified well, were further used for real-time PCR quantification. In order to overcome the effects of any remaining inhibitors that are not eliminated during RNA and mRNA extraction, Bovine serum albumin (BSA) was added for all PCR assays to provide some resistance to inhibitors during the PCR step. The choice of DNA polymerases which can have a large impact on resistance to inhibition was extensively tested, and DNA polymerases which are among the available choices the most sensitive to inhibition were selected for further analysis.

**Table 1:** **Primers used in this study**.

Target	Primer	Sequence (5′-3′)	Reference
Geobacteraceae-specific citrate synthase (*gltA*)	CS375nF	AACAAGATGRCMGCCTGGG	Akob et al. (2008)
	CS598nR	TCRTGGTCGGARTGGAGAAT	
	CS18nF	CTCGCGRCATYCGCAGTCT	This study, modified from Holmes et al. (2005)
	CS821nR	TGRCCGGCRTTCAGGGTAT	
	CS1Fdeg	CCGYGACATYCGCWGYCT	
	CS2Rdeg	TGRCCGGMRTTCAGGGTAT	
Dissimilatory sulfite reductase (*dsrA*)	DSR1F	ACSCACTGGAAGCACG	Wagner et al. (1998)
	DSR4R	GTGTAGCAGTTACCGCA	
	DSRQP3R	CGCATGGTRTGRAARTG	This study
	DSR2MF	CTGGAARGAYGACATCAA	This study, modified from Wagner et al. (1998)
Sulfate-reducing bacteria-specific 16S rRNA	DSV230f	GRGYCYGCGTYYCATTAGC	Daly et al. (2000)
	DSV838r	SYCCGRCAYCTAGYRTYCATC	

### Reverse transcription PCR and real-time PCR quantification of mRNA transcripts

cDNA synthesis was performed with *dsrA*-specific, *gltA*-specific, and SRB-specific 16S rRNA reverse primers, 0.5 μg template mRNA or total RNA, and MultiScribe™ MuLV reverse transcriptase (200 U; Applied Biosystems, Foster City, CA, USA) incubated at 25°C for 20 min followed by at 37°C for 120 min, and enzyme inactivation at 80°C for 5 s.

The cDNAs were amplified with gene-specific primers and the resulting amplicons were purified. The purified *dsrA* or *gltA* RT-PCR amplicons were quantified and prepared for serial dilution, which were used as calibration standards for the real-time PCR quantification (qPCR), and stored at −20°C. The detection limits of PCR assays were determined from three independent measurements as previously described (Chin et al., 2004). All assays had a minimum sensitivity of 10^0^–10^1^ target molecules per reaction. The precision and reproducibility of quantification were carefully optimized, and correct lengths of PCR products were verified (Chin et al., 2004). The cDNA which was generated with *dsrA*-specific or *gltA*-specific primers was quantified with real-time quantitative PCR, using SYBR Green. All reactions were carried out in 20 μl reaction volume containing 0.02 units of iProof High Fidelity polymerase (BioRad, Hercules, CA, USA), buffer, 2.5 pmol each dNTP, bovine serum albumin, and 20 pmol of each primer pair. The temperature profile was composed of an initial activation step at 50°C for 2 min and denaturation at 98°C for 30 s, followed by 40 cycles of denaturation at 98°C for 10 s, annealing at 52 (*gltA*) or 53°C (*dsrA*) for 2 s, and elongation at 65°C for 32 s, with a final extension step at 65°C for 6 min. In order to prevent primer dimer formation during qPCR, optimal primer pairs were designed and appropriate primer concentrations were used. The primer optimization matrix was performed with different amounts of cDNA to exclude concentration-depending phenomena, and qPCR assays were performed with controls without template (NTCs) followed by a dissociation curve to check for primer dimers and non-specific products. If more than a single peak was observed on the dissociation curve, the concentration of primer and MgCl_2_ was optimized. An amount of primer and MgCl_2_ that forms no primer dimers and gives optimal amplification was used for qPCR assays of all the samples. qPCR analysis of the cDNA was carried out with the Applied Biosystems 7500 Real-Time PCR system using 7500 Real-Time PCR System Sequence Detection Software (Version 1.3.1).

### Cloning, sequencing, and phylogenetic analysis

Six libraries were generated with the *dsrA*- or *gltA*-specific RT-PCR amplicons of short fragment (116 and 224 bp lengths amplified with primers DSR1F/DSRQP3R and CS375nF/CS598nR, respectively) obtained from mRNA, which were isolated from microcosm, Area 2, and Area 3 borehole sediments, respectively, to confirm the specificity of the real-time PCR products. Three libraries were generated with *gltA*-specific RT-PCR amplicons of long fragments (ca. 807–891 bp lengths, amplified with primers CS18nF/CS821nR or CS1Fdeg/CS2Rdeg) for phylogenetic analysis. Additionally, one library was generated with *dsrAB* gene amplicons of long fragments obtained from DNA isolated from microcosm sediment (ca. 1.4 kb length, amplified with primers DSR2MF/DSR4R), and another library was generated with SRB-specific 16S rRNA RT-PCR amplicons (ca. 610 bp length, amplified with primers DSV230f/DSV838r) obtained from total RNA isolated from Area 2 borehole sediment for phylogenetic analyses. Amplicons were cloned in to the pCR2.1-TOPO vector with the One Shot TOPO TA cloning kit or pCR4Blunt-TOPO vector with the Zero Blunt TOPO PCR Cloning Kit (Invitrogen). A total of 30 clones were randomly selected from each *gltA* mRNA library, SRB-specific 16S rRNA library, and *dsrAB* gene library, and plasmid inserts were sequenced with M13 primers using an ABI 3730xl DNA Analyzer. Sequences were compared to the GenBank database using the BLAST program (Altschul et al., 1990). Phylogenetic analysis was performed using the ARB software package (Ludwig et al., 2004). The ARB_EDIT tool was used for automatic sequence alignment, and the sequences were checked and corrected manually. Trees were calculated from aligned nucleotide and deduced *gltA* amino acid sequences with the ARB software package, using neighbor-joining, FITCH, and maximum likelihood methods. Trees constructed with amino acid and nucleotide sequences yielded similar results. Tree topology was constructed from nucleotide sequences using neighbor-joining analysis with Jukes–Cantor distance correction method and verified by maximum likelihood algorithm.

### Nucleotide sequence accession numbers

The nucleotide sequences of *dsr*, *gltA*, and 16S rRNA genes retrieved in this study were deposited in the EMBL database under the accession numbers HE856492–HE856617.

## Results

### Expression of *dsr*A and *glt*A during biostimulation

**Figure 1:**
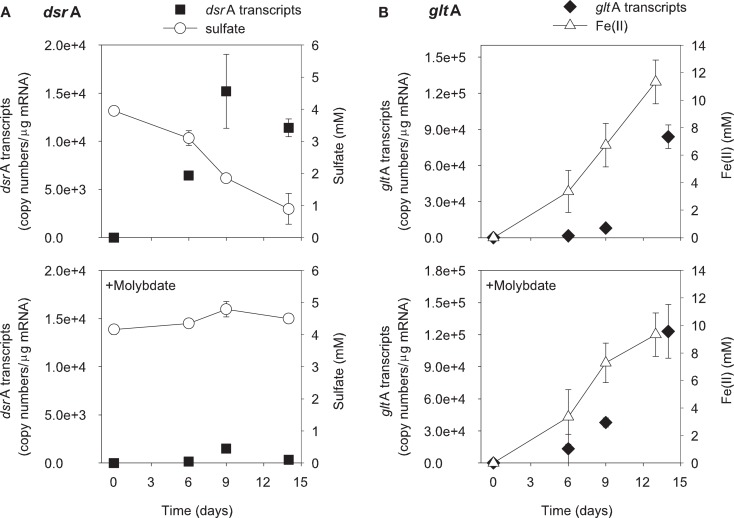
**Expression of *dsr*A (A) and *glt*A (B) genes related to electron acceptor reduction in ethanol-biostimulated microcosms**. The lower panels show treatments amended with molybdate as an inhibitor of sulfate reduction. Data are means ± standard deviations of triplicate determinations on a pooled sample from triplicate microcosms.

**Figure 2:**
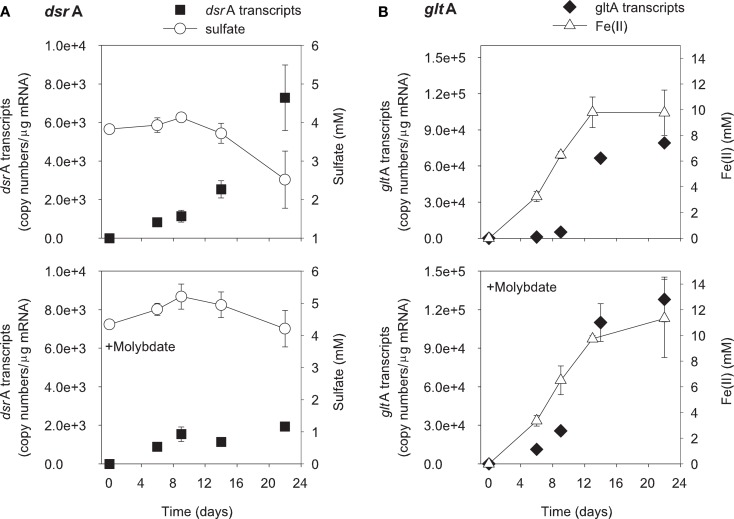
**Expression of *dsr*A (A) and *glt*A (B) genes related to electron acceptor reduction in acetate-biostimulated microcosms**. The lower panels show treatments amended with molybdate as an inhibitor of sulfate reduction. Data are means ± standard deviations of triplicate determinations on a pooled sample from triplicate microcosms.

The addition of ethanol or acetate stimulated sulfate reduction only in treatments without molybdate, whereas Fe(III) reduction [accumulation of Fe(II)] was observed in all treatments (Figures 1 and 2). *DsrA* transcript levels correlated with sulfate reduction activity in the treatments amended with carbon substrates (Figures 1A and 2A). In the ethanol-amended treatment, *dsrA* transcripts increased linearly to a maximum of 1.52 × 10^4^ copies per μg mRNA as ∼3.2 mM sulfate was reduced at day 9 and transcript levels decreased as sulfate concentrations were depleted to below 2 mM (Figure 1A). In the acetate-amended treatment, *dsrA* transcript levels were lower with 7.29 × 10^3^ copies per μg mRNA detected in parallel with the depletion of 1.3 mM sulfate at day 22 when sulfate reduction ceased (Figure 2A). In treatments where molybdate inhibited sulfate reduction (Figures 1A and 2A), *dsrA* transcripts were detected at a relatively low level (<2.00 × 10^3^ copies per μg mRNA), and little to no change was observed with time.

Levels of *Geobacteraceae*-specific *gltA* transcripts correlated with Fe(III) reduction in both ethanol- and acetate-amended treatments (Figures 1B and 2B). The majority of Fe(II) accumulated between days 5 and 8 and the addition of molybdate did not impact the amount of Fe(II) produced or the level of transcripts (Figures 1B and 2B). In the ethanol only treatment at day 22, 8.4 × 10^4^ *gltA* transcript copies per μg mRNA were observed, whereas 1.2 × 10^5^ *gltA* copies per μg mRNA were observed on the same day in the ethanol with molybdate treatment (Figure 1B). Approximately 9.8 and 9.6 mM Fe(II) accumulated and the highest numbers of *gltA* transcripts were 8.4 × 10^4^ and 1.2 × 10^5^ (copies per μg mRNA) in the acetate and acetate with molybdate treatments, respectively (Figure 2B).

**Figure 3:**
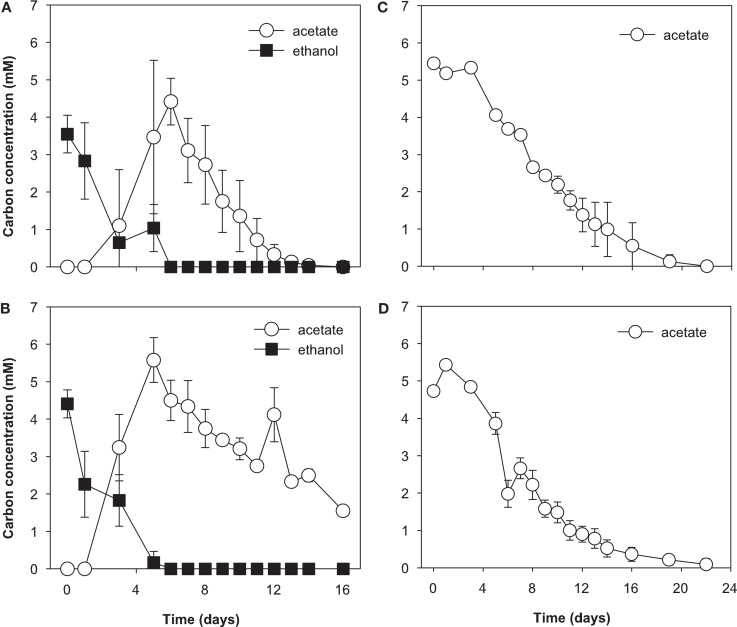
**Electron donor utilization biostimulated microcosms**. **(A)** Ethanol only treatment, **(B)** ethanol with molybdate, **(C)** acetate-only, and **(D)** acetate with molybdate; molybdate was added as a sulfate reduction inhibitor. Data are means ± standard deviations of triplicate microcosms.

During the initial 5 days of incubation, U(VI) concentrations decreased in all treatments, however, a more substantial decrease was observed in ethanol treatments (data not shown). U(VI) concentrations increased in conjunction with Fe(III) reduction and subsequently decreased by day 16 in the all treatments. Approximately 12 and 5 μM U(VI) was removed from solution in the ethanol and acetate treatments, respectively. Ethanol was consumed from days 0 to 6 in ethanol-amended treatments and was incompletely oxidized to acetate (Figures 3A and 3B). Acetate accumulated until day 6 and then was slowly consumed for the remainder of the incubation. In acetate-amended treatments, acetate was consumed starting on day 4 and ceased on day 22 of the incubation (Figures 3C and 3D).

### *In situ* metabolic activity of SRB and FeRB in contaminated sediments

**Figure 4:**
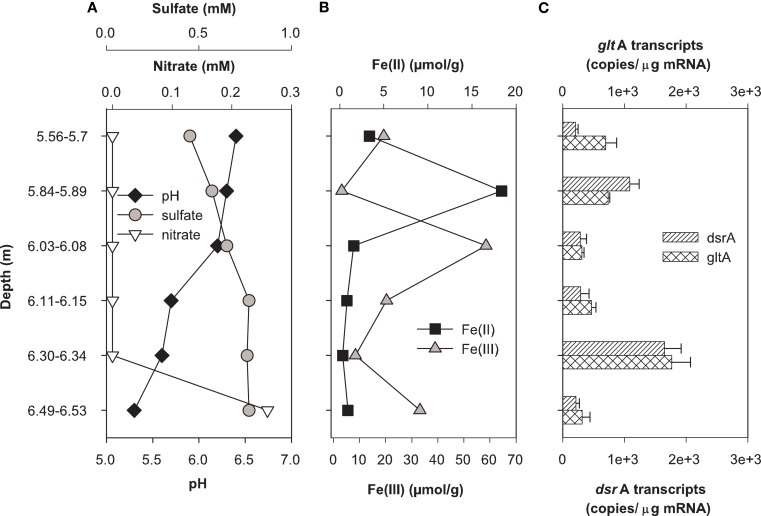
**Geochemistry (A,B) and levels of *dsr*A and *glt*A transcripts (C) in the depth interval from 1.23 to 15.08 m of Area 2 borehole FB107**. Values for transcript levels are means ± standard deviations of triplicate determinations on a pooled sample from triplicate borehole sediment samples. Nitrate, sulfate, pH, and iron data were reported previously as supplemental material in Green et al. (2010). Uranium was not analyzed.

**Figure 5:**
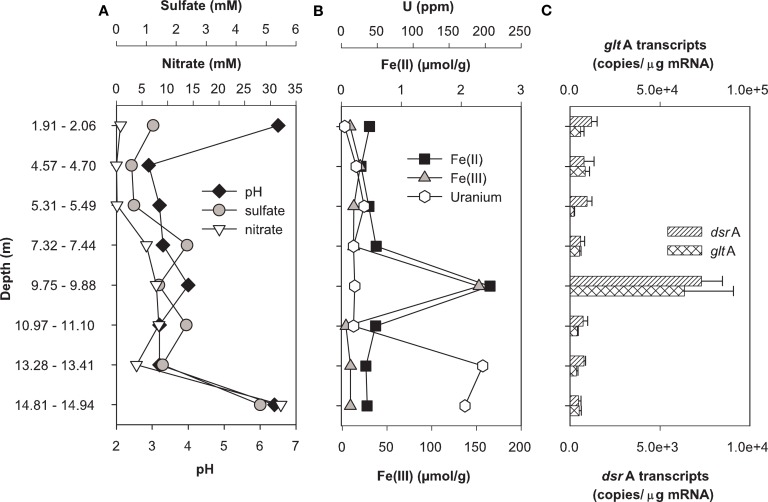
**Geochemistry (A,B) and levels of *dsr*A and *glt*A transcripts (C) in the depth interval from 5 to 7 m of Area 3 borehole FB124**. Data for transcript levels are means ± standard deviations of triplicate determinations on a pooled sample from triplicate borehole sediment samples. Nitrate, sulfate, pH, iron, and uranium data were reported previously as supplemental material in Green et al. (2010).

Expression of *dsrA* and *gltA* were quantified in parallel with the determination of the most abundant electron acceptors present in core samples from uranium-contaminated subsurface sediments (nitrate, sulfate, iron, uranium). Significant transcripts were detected in all sediments sampled from the OR-IFRC. Overall, transcript levels were variable and approached the limit of detection from subsurface regions exposed to moderate (FB107, Figure 4) and high levels (FB124, Figure 5) of uranium contamination. In borehole FB124, from the highly contaminated Area 3 of the ORFRC, expression of *dsr*A and *glt*A transcripts and the geochemistry indicated ongoing microbial activity in a depth of 9.75–9.88 m below surface (Figure 5). The highest level of *dsr*A and *glt*A transcripts were observed in this depth interval along with low sulfate and high Fe(II) concentrations. The other depth intervals of FB124 had a low level of gene expression and little geochemical evidence of sulfate or iron reduction. In contrast, depth profiling of gene expression and geochemistry in FB107 did not provide clear evidence for *in situ* microbial activity (Figure 4). In this core, the level of transcripts was much lower than in FB124 (Figures 4C and 5C, note different scales) and the depth interval (6.30–6.34 m below surface) with the highest level of transcripts did not have high Fe(II) or low sulfate concentration (Figure 4). The region with the highest Fe(II) concentration (5.84–5.89 m below surface) had moderate expression of *glt*A. Although nitrate concentrations were not elevated in sediments from FB107 (Figure 4), groundwater sampled from nearby wells has routinely shown nitrate concentrations in the 2 mM range (Akob et al., 2008; Green et al., 2010, 2012). Thus, though active Fe(III) and sulfate reducers were detected, their metabolism was likely inhibited by the presence of substantial concentrations of the competing electron acceptor, nitrate.

### Phylogeny of active SRB and FeRB during biostimulation and in *in situ* contaminated sediments

**Figure 6:**
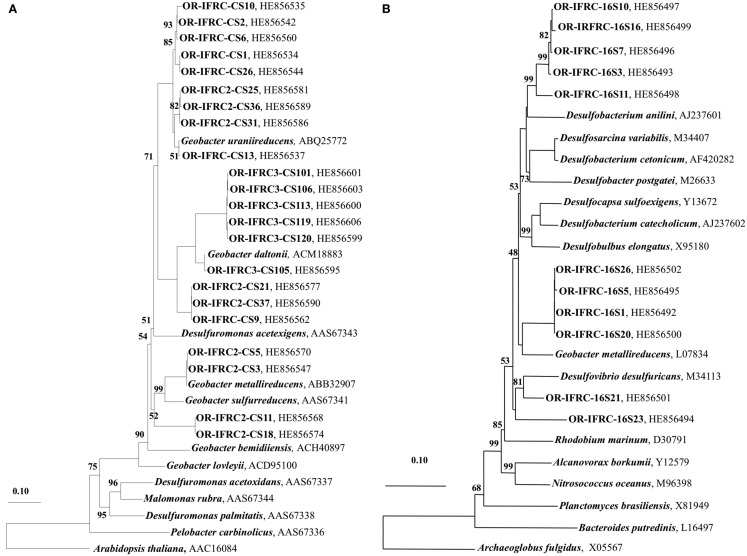
**(A)** Phylogenetic tree indicating the relationship of selected citrate synthase gene (*gltA*) mRNA sequences retrieved from OR-IFRC Area 2 sediment ethanol-amended microcosms (OR-IFRC), Area 2 *in situ* borehole sediments (OR-IFRC2), and Area 3 *in situ* borehole sediments (OR-IFRC3) and **(B)** Phylogenetic tree indicating the relationship of selected sulfate-reducing bacteria-specific 16S rRNA sequences retrieved from OR-IFRC Area 2 *in situ* borehole sediments (OR-IFRC16S). The scale bar indicates the estimated number of base changes per nucleotide sequence position. The accession numbers of the sequences are indicated.

The phylogeny of active sulfate and iron-reducers during biostimulation and in *in situ* contaminated sediments was evaluated. Phylogenetic analyses of Geobacteraceae*-*specific *gltA* mRNA sequences (∼891 bp length) retrieved from Area 2 ethanol-amended microcosms demonstrated that groups closely related to *G. uraniireducens* and *G*. *daltonii* FRC-32 are metabolically active and abundant (Figure 6A). BLAST analysis of short *gltA* mRNA sequences (224 bp length) confirmed the specificity of qPCR amplicons, and also suggested that groups closely related to *Geobacter* species are active and abundant in Area 2 sediments (Table 3). No amplicons were obtained from mRNA or DNA extracted from OR-IFRC sediments with the primer pair DSR1F/DSR4R, which previously recovered *dsrAB* DNA sequences from many other environments and amplified the *dsrAB* genes from a pure culture of *Desulfovibrio desulfuricans* in this study. However, the primer pair DSR2MF/DSR4R, which amplifies a smaller (1.4 kbp) fragment of *dsrAB* in both Bacteria and Archaea, amplified portions of the *dsr*AB sequences from DNA but not from mRNA extracted from ethanol-amended microcosms. BLAST analysis of *dsrAB* DNA sequences (∼850 bp length) demonstrated the presence of groups belonging to Desulfovibrionaceae (clones OR-IFRC-DSRMd-16 and OR-IFRC-DSRMd-48) and Desulfobacteraceae (clones OR-IFRC-DSRMd-19 and OR-IFRC-DSRMd-39) in Area 2 microcosm sediments. Clone OR-IFRC-DSRMd-16 had 79% sequence similarity to *dsrAB* genes of *Desulfovibrio aminophilus* strain DSM 12254 (AY626029), whereas, clone OR-IFRC-DSRMd-48 was 99% related to *dsrAB* genes of *D. desulfuricans* (AJ249777). The Desulfobacteraceae clones OR-IFRC-DSRMd-19 and OR-IFRC-DSRMd-39 were related to dsrAB genes of *Desulfobacterium cetonicum* (77%, AF420282) and uncultured prokaryote clone DB_dsr12 (84%, EU350969), respectively. BLAST analysis of short *dsr*A mRNA sequences (116 bp length) retrieved from ethanol-amended microcosms confirmed the specificity of qPCR amplicons, and also suggested that groups closely related to *Desulfovibrio* species are metabolically active and abundant in biostimulated Area 2 sediments (Table 2).

**Table 2:** **Partial *dsr*A mRNA sequences retrieved from OR-IFRC ethanol-amended microcosms and *in situ* sediment**.

Source	Sequence designation	Closest relative (accession No.)	% Identity
Area 2 ethanol-amended sediment microcosms	OR-IFRC-DSRsM1-6	*Desulfovibrio carbinolicus* strain DSM 3852 dsrAB genes (AY626026)	85
	OR-IFRC-DSRsM2-12	*Desulfovibrio desulfuricans* isolate SRDQC dsrAB genes (DQ450464)	96
	OR-IFRC-DSRsM2-26	*Desulfovibrio desulfuricans* strain F28-1 dsrAB genes (DQ092635)	94
	OR-IFRC-DSRsM1-4	Uncultured prokaryote clone dsrSbI-66 dsrAB genes (AY167480)	92
	OR-IFRC-DSRsM1-3	Uncultured sulfate-reducing bacterium dsrA gene clone LSsed06Jan-005 (AB281007)	86
Area 2 *in situ* sediments	OR-IFRC-DSRsA2-12	*Desulfovibrio africanus* strain SR-1 (EU716165)	84
	OR-IFRC-DSRsA2-4	*Desulfovibrio carbinolicus* strain DSM 3852 dsrAB genes (AY626026)	87
	OR-IFRC-DSRsA2-7	*Desulfovibrio desulfuricans* subsp. *desulfuricans* dsrA gene (CAC09930)	94
	OR-IFRC-DSRsA2-8	*Desulfovibrio desulfuricans* subsp. *desulfuricans* dsrA gene (CAC09930)	94
	OR-IFRC-DSRsA2-10	Uncultured sulfate-reducing bacterium dsrA gene (BAF56324)	97
Area 3 *in situ* sediments	OR-IFRC-DSRsA3-1	*Desulfotomaculum thermocisternum* *dsr*A gene (AAC96107)	84
	OR-IFRC-DSRsA3-2	*Desulfovibrio desulfuricans* subsp. *desulfuricans* *dsr*A gene (CAC09930)	97
	OR-IFRC-DSRsA3-7	*Desulfovibrio desulfuricans* subsp. *desulfuricans* *dsr*A gene (CAC09930)	97
	OR-IFRC-DSRsA3-3	*Desulfovibrio simplex* *dsrA* gene (AAB66716)	97
	OR-IFRC-DSRsA3-5	Uncultured sulfate-reducing bacterium *dsrA* gene (CAJ47296)	97

*Sequence length is 116 bp and all sequences were related to members of the Deltaproteobacteria*.

Phylogenetic analyses of *gltA* mRNA sequences (∼891 bp length) retrieved from *in situ* Area 2 borehole sediments demonstrated that groups closely related to *G. uraniireducens*, *G*. *daltonii* FRC-32, and *G. metallireducens* are metabolically active and abundant *in situ* (Figure 6A). This result corresponds with the result obtained in the Area 2 microcosm study, and also indicates a higher diversity than in ethanol-amended microcosms. BLAST analysis of short *gltA* mRNA sequences (224 bp length) confirmed the specificity of qPCR amplicons, and also suggested that groups closely related to *Geobacter* species are active and abundant in the Area 2 sediments (Table 3). BLAST analysis of short *dsrA* mRNA sequences (116 bp length) retrieved from Area 2 *in situ* sediments confirmed the specificity of qPCR amplicons, and also suggested that groups closely related to *Desulfovibrio* species are active and abundant *in situ* (Table 2). In addition, phylogenetic analysis of SRB-specific 16S rRNA sequences (∼610 bp length) confirmed the abundance and activity of groups belonging to *Geobacteraceae*, *Desulfovibrionaceae*, and *Desulfobacteraceae* in Area 2 *in situ* sediments (Figure 6B).

**Table 3:** **Partial *glt*A mRNA sequences retrieved from OR-IFRC ethanol-amended microcosms and *in situ* sediment**.

Source	Sequence designation	Closest relative (accession No.)	% Identity
Area 2 ethanol-amended sediment microcosms	OR-IFRC-CSsM2-2	*Geobacter uraniireducens* Rf4 citrate synthase (*gltA*) gene (CP000698)	84
	OR-IFRC-CSsM2-4	Uncultured *Geobacteraceae* clone CS9-11 citrate synthase (*gltA*) gene (EU352200)	92
	OR-IFRC-CSsM2-6	Uncultured *Geobacteraceae* clone CS9-11 citrate synthase (*gltA*) gene (EU352200)	87
	OR-IFRC-CSsM2-7	*Geobacter bemidjiensis* Bem, citrate synthase (*gltA*) gene (CP001124)	83
	OR-IFRC-CSsM2-8	Uncultured *Geobacteraceae* clone CS9-11 citrate synthase (*gltA*) gene (EU352200)	97
Area 2 *in situ* sediments	OR-IFRC-CSsA2-3	*Geobacter uraniireducens* Rf4 citrate synthase (*gltA*) gene (CP000698)	84
	OR-IFRC-CSsA2-4	*Geobacter bemidjiensis* Bem, citrate synthase (*gltA*) gene (CP001124)	84
	OR-IFRC-CSsA2-6	*Geobacter bemidjiensis* Bem, citrate synthase (*gltA*) gene (CP001124)	85
	OR-IFRC-CSsA2-7	*Geobacter bemidjiensis* Bem, citrate synthase (*gltA*) gene (CP001124)	83
	OR-IFRC-CSsA2-8	*Geobacter uraniireducens* Rf4 citrate synthase (*gltA*) gene (CP000698)	84
Area 3 *in situ* sediments	OR-IFRC-CSsA3-20	*Geobacter daltonii* FRC-32 citrate synthase (*glt*A) gene (YP_002535984)	81
	OR-IFRC-CSsA3-23	*Geobacter daltonii* FRC-32, citrate synthase (*glt*A) gene (YP_002535984)	84
	OR-IFRC-CSsA3-26	*Geobacter daltonii* FRC-32, citrate synthase (*glt*A) gene (YP_002535984)	84
	OR-IFRC-CSsA3-27	*Geobacter daltonii* FRC-32 citrate synthase (*glt*A) gene (YP_002535984)	81
	OR-IFRC-CSsA3-29	*Geobacter daltonii* FRC-32, citrate synthase (*glt*A) gene (YP_002535984)	84

*Sequence length is 224 bp and all sequences were related to members of the Deltaproteobacteria*.

Phylogenetic analyses of *gltA* mRNA sequences (∼891 bp length) retrieved from *in situ* Area 3 borehole sediments demonstrated that only a group closely related to *G. daltonii* is active and dominant (Figure 6A). BLAST analysis of short *gltA* mRNA sequences confirmed the specificity of qPCR amplicons, and also suggested that a group closely related to *G. daltonii* is active and abundant in Area 3 sediments (Table 3). BLAST analysis of short *dsrA* mRNA sequences (116 bp length) retrieved from Area 3 *in situ* borehole sediments confirmed the specificity of qPCR amplicons, and also suggested that groups closely related to *Desulfovibrio* and *Desulfotomaculum* species are active and abundant in these subsurface sediments (Table 2).

## Discussion

### Metabolic activity of FeRB and SRB in uranium-contaminated subsurface sediments

Bioremediation, via biostimulation of microbial communities by addition of large quantities of electron donor, is a proposed strategy for uranium immobilization at many uranium-contaminated sites managed by the U.S. DOE. The addition of electron donors, e.g., ethanol and acetate, has been shown previously to stimulate the reductive removal of U(VI) in the subsurface of many of these sites (e.g., Anderson et al., 2003; Wu et al., 2006; Akob et al., 2008; Michalsen et al., 2009). Iron(III)-reducing bacteria (FeRB) and SRB have been identified as the functional guilds of microorganisms likely to catalyze U(VI) reduction during *in situ* bioremediation experiments conducted at the DOE’s OR-IFRC site or the Old Rifle site in Colorado, in particular (Kostka and Green, 2011). Although a large number of studies have shown that the abundance of these microbial groups increases in response to electron donor addition and under metal-reducing conditions, the activity of specific metal-reducing bacterial populations (e.g., SRB and FeRB) remains difficult to assess under *in situ* conditions.

Biogeochemical methods for quantifying rates of sulfate or Fe(III) reduction (Canfield et al., 2005) are tedious and time consuming and thus cannot be easily applied over the scales necessary for monitoring *in situ* bioremediation. Thus, a quantitative molecular approach for the determination of Fe(III) or sulfate reduction activity would aid in diagnosing the success of bioremediation strategies along with *in situ* controls of the enzymatically catalyzed processes. A method that focuses on the gene expression of metal-reducing bacterial populations would be ideal since the active populations could be identified along with the quantification of activity. The desired target should be a phylogenetically informative gene that is highly conserved and unique to a distinct group and for which expression patterns are correlated to metabolic rates. The dissimilatory (bi)sulfite reductase (*dsrAB*) gene provides such a target for SRB since it is highly conserved and codes for the enzyme responsible for the rate-limiting step of sulfate reduction. In the case of FeRB, such a robust gene target has not been identified because numerous pathways exist for metal respiration that involved a number of different proteins, which are poorly conserved (DiChristina, 2005a).

The expression of respiratory genes involved in Fe(III) reduction was shown to correlate with rates of metabolism in pure cultures of the *Geobacteraceae* (Chin et al., 2004). However, transcript level of respiratory genes was shown to respond to other parameters besides rates of metabolism and growth (Chin et al., 2004). It was proposed that the expression of genes linked to central carbon metabolism may provide an alternative proxy for metabolic rates of Fe(III)-reducing members of the *Geobacteraceae* (Holmes et al., 2005). *Geobacter* species are known to predominate under Fe(III)-reducing conditions (Bond et al., 2005; Holmes et al., 2005) and are in fact capable of outcompeting other FeRB in Fe(III)-rich environments (Rooney-Verga et al., 1999; Snoeyenbos-West et al., 2000; Röling et al., 2001). In addition, *Geobacteraceae* often predominate in uranium-contaminated subsurface sediments undergoing bioremediation, including the above mentioned DOE sites at Oak Ridge, TN, USA and in Rifle, CO, USA (Holmes et al., 2002, 2007; Anderson et al., 2003; North et al., 2004). Thus in this study, we targeted the citrate synthase (*gltA*) gene that is unique to the *Geobacteraceae* and encodes for an enzyme involved in the incorporation of acetate in to the TCA cycle (Bond et al., 2005; Holmes et al., 2005). To date, a total of 16 *Geobacter* species were isolated that are all capable of Fe(III) reduction (Lovley et al., 2004; Nevin et al., 2005, 2007; Sung et al., 2006; Shelobolina et al., 2007, 2008; Prakash et al., 2010). Eight of these isolates, *Geobacter daltonii, G. uraniireducens, G. metallireducens, G. sulfurreducens, G. psychrophilus, G. bemidjiensis, G. lovleyii*, and *G. thiogenes*, have citrate synthase genes available on the NCBI sequence database that are unique as they are eukaryote-like (Methe et al., 2003; Bond et al., 2005) distinguishing them from other prokaryotic FeRB and heterotrophs. The other isolates currently do not have genome sequences available so the genes have not been identified, but their similar metabolisms supports the inference that they may harbor the same, conserved citrate synthase gene. It is important to note that many organisms that are closely related to the *Geobacteraceae* do not possess a complete citric acid cycle or citrate synthase or have citrate synthase genes that are prokaryote-like instead of eukaryote-like. In addition, a closely related sulfate-reducer *Desulfovibrio desulfuricans* lacks citrate synthase in its genome, which was suggested by Bond et al. (2005) to indicate that citrate synthase was an important requirement in the evolution of *Geobacteraceae* to reduce Fe(III). We are aware that citrate synthase is not a molecular marker specifically for Fe(III) reduction, however it has been shown that its expression in pure cultures correlated directly with the central metabolism that was required for electron transfer to Fe(III) (Holmes et al., 2005).

Here we show for the first time that gene expression quantified as transcript levels of the *Geobacteraceae* clade of FeRB directly correlates with process rates and we identify specific microbial groups that are likely to catalyze metal reduction *in situ*. The competition between Fe(III) and sulfate reducers for carbon substrates could also be observed. In general, transcript levels of *dsrA* and *Geobacteraceae*-specific *gltA* paralleled with the extent of sulfate and Fe(III) reduction, respectively, in all of our incubations. In the acetate-amended treatments, electron-accepting processes occurred according to thermodynamic predictions (Canfield et al., 2005). The bulk of the more energetically favorable process, Fe(III) reduction, occurred during the first 2 weeks of incubation, whereas most sulfate reduction occurred after 2 weeks. The rapid reduction of Fe(III) in acetate treatments was correlated with an increase in the expression of the *Geobacteraceae*-specific *gltA* gene, which indicates an increase in the growth and metabolism of members of the *Geobacteraceae* family. A recent study reported that *Geobacter* spp. grow rapidly after the addition of acetate to uranium-contaminated sediments and that in conditions of excess electron donor their abundance is primarily controlled by the availability of microbially reducible Fe(III) (Barlett et al., 2012). In our microcosms amended with acetate and molybdate, we observed higher expression of *gltA* compared to the acetate treatment without molybdate. It is likely that this increase in gene expression in the presence of molybdate reflects the competition for electron donors between FeRB and SRB. With the addition of molybdate, the activity of SRB was depressed thereby removing competition for added acetate. This conclusion is supported by our observation that acetate was consumed at a faster rate in the presence of molybdate. In contrast to Barlett et al. (2012), evidence from this study does not support the concurrent growth of FeRB and SRB in the presence of excess acetate. Rather, we conclude that in addition to Fe(III) availability, competition between FeRB and SRB is a key factor in limiting the activity of *Geobacter* during uranium bioremediation.

The choice of electron donor for bioremediation may directly impact U(VI) biotransformation by affecting the competition between FeRB and SRB. During OR-IFRC bioremediation experiments with ethanol, Fe(III) and sulfate reduction occurred simultaneously and SRB were more abundant than FeRB, suggesting that SRB play a more important role in U(VI) immobilization (Wu et al., 2007; Akob et al., 2008; Cardenas et al., 2008; Hwang et al., 2009). In corroboration of previous work, we observed concurrent Fe(III) and sulfate reduction in ethanol-amended treatments, which goes against thermodynamic predictions (Canfield et al., 2005). The overlap in activity suggests that separate populations of Fe(III) and sulfate reducers were successfully competing for ethanol while reducing their preferred electron acceptor. It may be that SRB populations that couple reduction to ethanol oxidation are incapable of using Fe(III) thereby making sulfate the more energetic electron acceptor for their metabolism. Ethanol was first oxidized incompletely to acetate in the initial 6–8 days of incubation and then acetate was subsequently utilized by microbial consortia. Acetate was completely consumed in the ethanol only treatments, while >1 mM acetate remained in the treatments amended with ethanol + molybdate. This likely indicates that the microcosms were depleted in Fe(III) and sulfate was not utilized due to the fact that sulfate reducers were inhibited by molybdate. Ethanol addition resulted in the enhanced removal of soluble U(VI) relative to acetate amendment.

Prior to the onset of active sulfate reduction, the levels of *dsrA* transcripts increased in electron donor-amended microcosms and when sulfate concentrations were depleted, levels of *dsr*A transcripts concurrently decreased. This is not surprising because *dsrAB* codes for the dissimilatory (bi)sulfite reductase enzyme which is responsible for the rate-limiting step of sulfate reduction (Wagner et al., 1998). The increased *dsrA* gene expression indicates the up regulation of the DSR operon, which is necessary for the cells to synthesize and to transport the enzymes needed to reduce the available sulfate. As expected in microcosms amended with molybdate to inhibit sulfate reduction the levels of *dsrA* transcripts were at the lower limit of detection. Unlike the response of *dsrA* transcripts, *Geobacteraceae*-specific *gltA* transcript levels did not increase prior to Fe(III) reduction but increased proportionally with Fe(III) reduction activity. This follows as *gltA* codes for an enzyme involved in the incorporation of acetate in to the TCA cycle (Bond et al., 2005; Holmes et al., 2005). Therefore, since *gltA* expression is not required for Fe(III) reduction but carbon metabolism, we would expect it to correlate more closely with acetate consumption. The increase in *glt*A expression indeed precedes consumption of acetate and indicates up regulation of the TCA cycle prior to acetate decrease. The quantification of *Geobacteraceae*-specific *glt*A transcripts verified a direct association between Fe(III) reduction and the oxidation of acetate.

### Phylogeny of active metal-reducing bacteria in uranium-contaminated sediments

Quantification of *gltA* and *dsrA* gene expression was successful in sediment samples from the moderately and highly contaminated OR-IFRC Areas 2 and 3, respectively. The observation that Geobacteraceae-related FeRB and SRB are metabolically active within borehole sediments without biostimulation provides evidence that these organisms are available to promote bioremediation and the long-term stability of reduced uranium *in situ*. The detection of transcripts in unamended subsurface sediments further demonstrates the sensitivity and specificity of the mRNA-based method. Previous studies found that Geobacteraceae*-*related FeRB and SRB were below detection in highly contaminated Area 3 of the OR-IFRC subsurface (Cardenas et al., 2008). We observed that transcript levels of *gltA* and *dsrA* were low and only correlated with the abundance of electron acceptors in Area 3 sediments. Lower transcript levels in Area 2 sediments indicated that FeRB and SRB are active only at background levels, therefore their activity is not directly reflected in the prevailing biogeochemistry of the surrounding sediment. However, we cannot ignore the possibility that transport and sampling of sediments affected microbial activity and biased transcript analysis. For Area 3 sediments the highest levels of expression were associated with sediment zones with higher Fe(II) content and low sulfate concentrations. The low sulfate concentrations and high *dsr*A transcript levels are indicative of on going sulfate reduction, whereas, *glt*A expression in regions with high Fe(II) concentrations indicated *in situ* activity of Geobacteraceae*-*related FeRB. The *in situ* activity of FeRB and SRB in Area 3 was surprising as this area is rich in nitrate, which serves as an alternative electron acceptor for FeRB and SRB, due to close proximity to the source zone, and is limited in electron donors or carbon substrates. However, some species of *Geobacter* are known to reduce nitrate, such as *G. humireducens* (Coates et al., 1998) and *G. metallireducens* (Lovley et al., 1993b), and some species are known to oxidize aromatic hydrocarbons (Prakash et al., 2010) which are present in the contaminant plume of Area 3 (http://www.esd.ornl.gov/orifrc/). Although the conditions in Area 3 are more favorable for nitrate-reducing bacteria, which are adapted to the high nitrate, low pH *in situ* conditions (Green et al., 2012), active FeRB and SRB populations are present and active at low levels *in situ*. Such subsurface zones with high *in situ* activity of FeRB and SRB are likely the areas best suited for *in situ* bioremediation, as active microbial populations could quickly respond to the input of supplemental electron donor.

Members of the *Geobacteraceae* family are often detected in conjunction with metal reduction in the uranium-contaminated subsurface during biostimulation with the addition of electron donors, such as ethanol (North et al., 2004; Akob et al., 2008; Mohanty et al., 2008; Burkhardt et al., 2010; Sitte et al., 2010; Vishnivetskaya et al., 2010; Van Nostrand et al., 2011). The majority of cultivation-independent studies in subsurface sediments were conducted at the DNA level with SSU rRNA gene targets. In this study, the results of the phylogenetic analysis of *gltA* mRNA sequences demonstrated that members of the *Geobacteraceae* are abundant and metabolically active in biostimulated subsurface sediments. Further, our results suggest that members of the subsurface *Geobacter* clade, closely related to *G. uraniireducens* and *G. daltonii*, are metabolically active iron reducers that mediate metal reduction in OR-IFRC subsurface sediments. *G. uraniireducens* and *G. daltonii* were isolated from U(VI)-contaminated subsurface environments at the Rifle and OR-IFRC sites, respectively (Shelobolina et al., 2008; Prakash et al., 2010), and these two species cluster with the phylogenetically coherent subsurface clade proposed by Holmes et al. (2007). Although these two *Geobacter* strains share 98.1% 16S rRNA gene sequence identity, their full genome sequences are highly divergent (Prakash et al., 2010). Limited physiological screening has begun to reveal substantial differences in electron acceptor and donor utilization within the subsurface clade of *Geobacter*. For example, *G. daltonii* and *G. toluenxydans* conserve energy for growth with aromatic contaminants as the electron donor, while *G. uraniireducens* does not (Kunapuli et al., 2010; Prakash et al., 2010). This may be explained by the fact that both *G. daltonii* and *G. toluenoxydans* were isolated from subsurface sediments contaminated with aromatic hydrocarbons, whereas *G. uraniireducens* was isolated from groundwater that was not substantially impacted by organic contaminants. A number of additional features may provide a competitive advantage to *Geobacter* in the subsurface including the ability to utilize acetate, chemotaxis, and nitrogen fixation (Childers et al., 2002; Holmes et al., 2004). Further quantitative analysis of *glt*A transcripts has the potential to aid in our understanding of the environmental controls of *Geobacteraceae*-mediated metal reduction. Primers designed for specific strains will likely reveal niche differentiation within the *Geobacter* family.

The active SRB observed during biostimulation and in intact core samples were related to members of the *Desulfovibrionaceae* and *Desulfobacteraceae* families within the *Deltaproteobacteria* and the *Clostridia* within the *Firmicutes*, and no sulfate-reducing Archaea were detected. This observation fits with previous studies at uranium-contaminated sites that correlated the activity of these organisms with the addition of carbon substrates (Chang et al., 2005; Akob et al., 2008; Burkhardt et al., 2010; Cardenas et al., 2010; Sitte et al., 2010; Vishnivetskaya et al., 2010; Miletto et al., 2011). The utilization of supplemental electron donors in our microcosms also fits with the known metabolism of the SRB detected, as ethanol is incompletely oxidized to acetate by *Desulfovibrio* and *Desulfotomaculum* species (Muyzer and Stams, 2008). In addition, both *Desulfovibrio* and *Desulfotomaculum* are known to enzymatically reduce U(VI) (Lovley and Phillips, 1992; Lovley et al., 1993a; Tebo and Obraztsova, 1998). This suggests that the activity of these organisms was directly related to the observed decrease in soluble uranium concentrations as was seen in earlier work (Akob et al., 2008; Cardenas et al., 2010; Vishnivetskaya et al., 2010; Van Nostrand et al., 2011). In environments co-contaminated with sulfate and nitrate such as the OR-IFRC, stimulation of *Desulfovibrio* may have a high bioremediation potential since the presence of sulfate represses nitrate reduction in this organism (Marietou et al., 2009). While members of the *Desulfobacteraceae* are known for acetate utilization and the complete oxidation of carbon substrates (Muyzer and Stams, 2008), these organisms have not been shown to reduce U(VI). Thus, it follows that we observed enhanced U(VI) immobilization under sulfate-reducing conditions when ethanol was used as the electron donor, whereas the linkage was not as strong when acetate was used.

## Conflict of Interest Statement

The authors declare that the research was conducted in the absence of any commercial or financial relationships that could be construed as a potential conflict of interest.

## References

[B1] Abdelouas A. Lu Y. Lutze W. Nuttall H. (1998). Reduction of U(VI) to U(IV) by indigenous bacteria in contaminated ground water. J. Contam. Hydrol. 35, 217–233 10.1016/S0169-7722(98)00134-X

[B2] Akob D. M. Mills H. J. Gihring T. M. Kerkhof L. Stucki J. W. Anastacio A. S. Chin K.-J. Küsel K. Palumbo A. V. Watson D. B. Kostka J. E. (2008). Functional diversity and electron donor dependence of microbial populations capable of U(VI) reduction in radionuclide-contaminated subsurface sediments. Appl. Environ. Microbiol. 74, 3159–3170 10.1128/AEM.02881-07 18378664 PMC2394950

[B3] Altschul S. F. Gish W. Miller W. Myers E. W. Lipman D. J. (1990). Basic local alignment search tool. J. Mol. Biol. 215, 403–410 10.1006/jmbi.1990.9999 2231712

[B4] Anderson R. T. Vrionis H. A. Ortiz-Bernad I. Resch C. T. Long P. E. Dayvault R. Karp K. Marutzky S. Metzler D. R. Peacock A. White D. C. Lowe M. Lovley D. R. (2003). Stimulating the in situ activity of *Geobacter* species to remove uranium from the groundwater of a uranium-contaminated aquifer. Appl. Environ. Microbiol. 69, 5884–5891 10.1128/AEM.69.10.5884-5891.2003 14532040 PMC201226

[B5] Barlett M. Zhuang K. Mahadevan R. Lovley D. (2012). Integrative analysis of *Geobacter* spp. and sulfate-reducing bacteria during uranium bioremediation. Biogeosciences 9, 1033–1040 10.5194/bg-9-1033-2012

[B6] Bond D. R. Mester T. Nesbo C. L. Izquierdo-Lopez A. V. Collart F. L. Lovley D. R. (2005). Characterization of citrate synthase from *Geobacter sulfurreducens* and evidence for a family of citrate synthases similar to those of eukaryotes throughout the Geobacteraceae. Appl. Environ. Microbiol. 71, 3858–3865 10.1128/AEM.71.7.3858-3865.2005 16000798 PMC1169064

[B7] Brina R. Miller A. G. (1992). Direct detection of trace levels of uranium by laser-induced kinetic phosphorimetry. Anal. Chem. 64, 1413–1418 10.1021/ac00037a020

[B8] Burkhardt E.-M. Akob D. M. Bischoff S. Sitte J. Kostka J. E. Banerjee D. Scheinost A. C. Küsel K. (2010). Impact of biostimulated redox processes on metal dynamics in an iron-rich creek soil of a former uranium mining area. Environ. Sci. Technol. 44, 177–183 10.1021/es902038e 19938814

[B9] Butler J. E. Young N. D. Lovley D. R. (2010). Evolution of electron transfer out of the cell: comparative genomics of six *Geobacter* genomes. BMC Genomics 11, 40 10.1186/1471-2164-11-40 20078895 PMC2825233

[B10] Canfield D. E. Kristensen E. Thamdrup B. (2005). Chapter 6: the iron and manganese cycles. Aquat. Geomicrobiol. 48, 1–65 10.1016/S0065-2881(05)48001-3

[B11] Cardenas E. Wu W.-M. Leigh M. B. Carley J. Carroll S. Gentry T. Luo J. Watson D. Gu B. Ginder-Vogel M. Kitanidis P. K. Jardine P. M. Zhou J. Criddle C. S. Marsh T. L. Tiedje J. M. (2008). Microbial communities in contaminated sediments, associated with bioremediation of uranium to submicromolar levels. Appl. Environ. Microbiol. 74, 3718–3729 10.1128/AEM.02308-07 18456853 PMC2446554

[B12] Cardenas E. Wu W.-M. Leigh M. B. Carley J. Carroll S. Gentry T. Luo J. Watson D. Gu B. Ginder-Vogel M. Kitanidis P. K. Jardine P. M. Zhou J. Criddle C. S. Marsh T. L. Tiedje J. M. (2010). Significant association between sulfate-reducing bacteria and uranium-reducing microbial communities as revealed by a combined massively parallel sequencing-indicator species approach. Appl. Environ. Microbiol. 76, 6778–6786 10.1128/AEM.01097-10 20729318 PMC2953039

[B13] Cataldo D. A. Haroon M. Schrader L. E. Youngs V. L. (1975). Rapid colorimetric determination of nitrate in plant tissue by nitration of salicylic acid. Commun. Soil Sci. Plant Anal. 6, 71–80 10.1080/00103627509366547

[B14] Chang Y. J. Long P. E. Geyer R. Peacock A. D. Resch C. T. Sublette K. Pfiffner S. Smithgall A. Anderson R. T. Vrionis H. A. Stephen J. R. Dayvault R. Ortiz-Bernad I. Lovley D. R. White D. C. (2005). Microbial incorporation of C-13-labeled acetate at the field scale: detection of microbes responsible for reduction of U(VI). Environ. Sci. Technol. 39, 9039–9048 10.1021/es040505f 16382923

[B15] Childers S. E. Ciufo S. Lovley D. R. (2002). *Geobacter metallireducens* accesses insoluble Fe(III) oxide by chemotaxis. Nature 416, 767–769 10.1038/416767a 11961561

[B16] Chin K.-J. Sharma M. Russell L. O’Neill K. Lovley D. (2008). Quantifying expression of a dissimilatory (bi)sulfite reductase gene in petroleum-contaminated marine harbor sediments. Microb. Ecol. 55, 489–499 10.1007/s00248-007-9294-2 17786505

[B17] Chin K.-J. Esteve-Nunez A. Leang C. Lovley D. R. (2004). Direct correlation between rates of anaerobic respiration and levels of mRNA for key respiratory genes in *Geobacter sulfurreducens*. Appl. Environ. Microbiol. 70, 5183–5189 10.1128/AEM.70.1.607-609.2004 15345398 PMC520918

[B18] Coates J. D. Ellis D. J. Blunt-Harris E. L. Gaw C. V. Roden E. E. Lovley D. R. (1998). Recovery of humic-reducing bacteria from a diversity of environments. Appl. Environ. Microbiol. 64, 1504–1509 9546186 10.1128/aem.64.4.1504-1509.1998 PMC106177

[B19] Daly K. Sharp R. J. McCarthy A. J. (2000). Development of oligonucleotide probes and PCR primers for detecting phylogenetic subgroups of sulfate-reducing bacteria. Microbiology 146, 1693–1705 10878133 10.1099/00221287-146-7-1693

[B20] DiChristina T. J. (2005a). Enzymology of electron transport: energy generation with geochemical consequences. Rev. Mineral. Geochem. 59, 27–52 10.2138/rmg.2005.59.3

[B21] DiChristina T. J. (2005b). New insights into the molecular mechanism of microbial metal respiration. Geochim. Cosmochim. Acta 69, A670

[B22] Finneran K. T. Anderson R. T. Nevin K. P. Lovley D. R. (2002). Potential for bioremediation of uranium-contaminated aquifers with microbial U(VI) reduction. Soil Sediment Contam. 11, 339–357 10.1080/20025891106781

[B23] Green S. J. Prakash O. Gihring T. M. Akob D. M. Jasrotia P. Jardine P. M. Watson D. B. Brown S. D. Palumbo A. V. Kostka J. E. (2010). Denitrifying bacteria isolated from terrestrial subsurface sediments exposed to mixed-waste contamination. Appl. Environ. Microbiol. 76, 3244–3254 10.1128/AEM.03069-09 20305024 PMC2869116

[B24] Green S. J. Prakash O. Jasrotia P. Overholt W. A. Cardenas E. Hubbard D. Tiedje J. M. Watson D. B. Schadt C. W. Brooks S. C. Kostka J. E. (2012). Denitrifying bacteria from the genus *Rhodanobacter* dominate bacterial communities in the highly contaminated subsurface of a nuclear legacy waste site. Appl. Environ. Microbiol. 78, 1039–1047 10.1128/AEM.06435-11 22179233 PMC3273022

[B25] Groudev S. Spasova I. Nicolova M. Georgiev P. (2010). In situ bioremediation of contaminated soils in uranium deposits. Hydrometallurgy 104, 518–523 10.1016/j.hydromet.2010.02.027

[B26] Holmes D. Nevin K. Lovley D. (2004). In situ expression of nifD in Geobacteraceae in subsurface sediments. Appl. Environ. Microbiol. 70, 7251–7259 10.1128/AEM.70.10.6023-6030.2004 15574924 PMC535187

[B27] Holmes D. E. Finneran K. T. O’Neil R. A. Lovley D. R. (2002). Enrichment of members of the family Geobacteraceae associated with stimulation of dissimilatory metal reduction in uranium-contaminated aquifer sediments. Appl. Environ. Microbiol. 68, 2300–2306 10.1128/AEM.68.5.2300-2306.2002 11976101 PMC127590

[B28] Holmes D. E. Nevin K. P. O’Neil R. A. Ward J. E. Adams L. A. Woodard T. L. Vrionis H. A. Lovley D. R. (2005). Potential for quantifying expression of the Geobacteraceae citrate synthase gene to assess the activity of Geobacteraceae in the subsurface and on current-harvesting electrodes. Appl. Environ. Microbiol. 71, 6870–6877 10.1128/AEM.71.11.6870-6877.2005 16269721 PMC1287699

[B29] Holmes D. E. O’Neil R. A. Vrionis H. A. N’Guessan L. A. Ortiz-Bernad I. Larrahondo M. J. Adams L. A. Ward J. A. Nicoll J. S. Nevin K. P. Chavan M. A. Johnson J. P. Long P. E. Lovley D. R. (2007). Subsurface clade of Geobacteraceae that predominates in a diversity of Fe(III)-reducing subsurface environments. ISME J. 1, 663–677 10.1038/ismej.2007.85 18059491

[B30] Hua B. Xu H. Terry J. Deng B. (2006). Kinetics of uranium(VI) reduction by hydrogen sulfide in anoxic aqueous systems. Environ. Sci. Technol. 40, 4666–4671 10.1021/es0519278 16913122

[B31] Hwang C. Wu W. Gentry T. J. Carley J. Corbin G. A. Carroll S. L. Watson D. B. Jardine P. M. Zhou J. Criddle C. S. Fields M. W. (2009). Bacterial community succession during in situ uranium bioremediation: spatial similarities along controlled flow paths. ISME J. 3, 47–64 10.1038/ismej.2008.123 18769457

[B32] Kostka J. E. Green S. J. (2011). “Microorganisms and processes linked to uranium reduction and immobilization,” in Microbial Metal and Metalloid Metabolism: Advances and Applications, eds Stolz J. F. Oremland R. S. (Washington, DC: ASM Press), 117–138

[B33] Kostka J. E. Luther G. W. (1994). Partitioning and speciation of solid-phase iron in salt-marsh sediments. Geochim. Cosmochim. Acta 58, 1701–1710 10.1016/0016-7037(94)90531-2

[B34] Kunapuli U. Jahn M. K. Lueders T. Geyer R. Heipieper H. J. Meckenstock R. U. (2010). Desulfitobacterium aromaticivorans sp. nov. and *Geobacter toluenoxydans* sp. nov., iron-reducing bacteria capable of anaerobic degradation of monoaromatic hydrocarbons. Int. J. Syst. Evol. Microbiol. 60, 686–695 10.1099/ijs.0.003525-0 19656942

[B35] Liger E. Charlet L. Van Cappellen P. (1999). Surface catalysis of uranium(VI) reduction by iron(II). Geochim. Cosmochim. Acta 63, 2939–2955 10.1016/S0016-7037(99)00265-3

[B36] Lovley D. Holmes D. Nevin K. (2004). Dissimilatory Fe(lII)and Mn(lV) reduction. Adv. Microb. Physiol. 49, 68 10.1016/S0065-2911(04)49005-5 15518832

[B37] Lovley D. Widman P. Woodward J. Phillips E. (1993a). Reduction of uranium by cytochrome c3 of *Desulfovibrio vulgaris*. Appl. Environ. Microbiol. 59, 3572–3576 8285665 10.1128/aem.59.11.3572-3576.1993 PMC182500

[B38] Lovley D. R. Giovannoni S. J. White D. C. Champine J. E. Phillips E. J. P. Gorby Y. A. Goodwin S. (1993b). *Geobacter metallireducens* gen. nov. sp. nov., a microorganism capable of coupling the complete oxidation of organic compounds to the reduction of iron and other metals. Arch. Microbiol. 159, 336–344 10.1007/BF00290916 8387263

[B39] Lovley D. R. Phillips E. J. (1992). Reduction of uranium by *Desulfovibrio desulfuricans*. Appl. Environ. Microbiol. 58, 850–856 1575486 10.1128/aem.58.3.850-856.1992 PMC195344

[B40] Ludwig W. Strunk O. Westram R. Richter L. Meier H. Yadhukumar Buchner A. Lai T. Steppi S. Jobb G. Forster W. Brettske I. Gerber S. Ginhart A. W. Gross O. Grumann S. Hermann S. Jost R. Konig A. Liss T. Lussmann R. May M. Nonhoff B. Reichel B. Strehlow R. Stamatakis A. Stuckmann N. Vilbig A. Lenke M. Ludwig T. Bode A. Schleifer K.-H. (2004). ARB: a software environment for sequence data. Nucleic Acids Res. 32, 1363–1371 10.1093/nar/gkh293 14985472 PMC390282

[B41] Marietou A. Griffiths L. Cole J. (2009). Preferential reduction of the thermodynamically less favorable electron acceptor, sulfate, by a nitrate-reducing strain of the sulfate-reducing bacterium *Desulfovibrio desulfuricans* 27774. J. Bacteriol. 191, 882–889 10.1128/JB.01171-08 19047345 PMC2632061

[B42] McLean E. O. (1982). “Soil pH and lime requirement,” in Methods of Soil Analysis: Part 2, Chemical and Microbiological Properties, 2nd Edn, eds Page A. L. Miller R. H. Keeney D. R. (Madison, WI: American Society of Agronomy), 199–209

[B43] Methe B. A. Nelson K. E. Eisen J. A. Paulsen I. T. Nelson W. Heidelberg J. F. Wu D. Wu M. Ward N. Beanan M. J. Dodson R. J. Madupu R. Brinkac L. M. Daugherty S. C. DeBoy R. T. Durkin A. S. Gwinn M. Kolonay J. F. Sullivan S. A. Haft D. H. Selengut J. Davidsen T. M. Zafar N. White O. Tran B. Romero C. Forberger H. A. Weidman J. Khouri H. Feldblyum T. V. Utterback T. R. Van Aken S. E. Lovley D. R. Fraser C. M. (2003). Genome of *Geobacter sulfurreducens*: metal reduction in subsurface environments. Science 302, 1967–1969 10.1126/science.1088727 14671304

[B44] Michalsen M. M. Peacock A. D. Smithgal A. N. White D. C. Spain A. M. Sanchez-Rosario Y. Krumholz L. R. Kelly S. D. Kemner K. M. McKinley J. Heald S. M. Bogle M. A. Watson D. B. Istok J. D. (2009). Treatment of nitric acid-, U(VI)-, and Tc(VII)-contaminated groundwater in intermediate-scale physical models of an in situ biobarrier. Environ. Sci. Technol. 43, 1952–1961 10.1021/es8012485 19368198

[B45] Miletto M. Williams K. H. N’Guessan A. L. Lovley D. R. (2011). Molecular analysis of the metabolic rates of discrete subsurface populations of sulfate reducers. Appl. Environ. Microbiol. 77, 6502–6509 10.1128/AEM.00576-11 21764959 PMC3187175

[B46] Mohanty S. R. Kollah B. Hedrick D. Peacock A. D. Kukkadapu R. K. Roden E. E. (2008). Biogeochemical processes in ethanol stimulated uranium-contaminated subsurface sediments. Environ. Sci. Technol. 42, 4384–4390 10.1021/es703082v 18605559

[B47] Muyzer G. Stams A. J. M. (2008). The ecology and biotechnology of sulphate-reducing bacteria. Nat. Rev. Microbiol. 6, 441–454 18461075 10.1038/nrmicro1892

[B48] Neretin L. N. Schippers A. Pernthaler A. Hamann K. Amann R. Jørgensen B. B. (2003). Quantification of dissimilatory (bi)sulphite reductase gene expression in *Desulfobacterium autotrophicum* using real-time RT-PCR. Environ. Microbiol. 5, 660–671 10.1046/j.1462-2920.2003.00452.x 12871233

[B49] Nevin K. P. Holmes D. E. Woodard T. L. Covalla S. F. Lovley D. R. (2007). Reclassification of *Trichlorobacter thiogenes* as *Geobacter thiogenes* comb. nov. Int. J. Syst. Evol. Microbiol. 57, 463–466 10.1099/ijs.0.63408-0 17329769

[B50] Nevin K. P. Holmes D. E. Woodard T. L. Hinlein E. S. Ostendorf D.W. Lovley D. R. (2005). *Geobacter bemidjiensis* sp. nov. and *Geobacter psychrophilus* sp. nov., two novel Fe(III)-reducing subsurface isolates. Int. J. Syst. Evol. Microbiol. 55, 1667–1674 10.1099/ijs.0.63417-0 16014499

[B51] North N. Dollhopf S. Petrie L. Istok J. Balkwill D. Kostka J. (2004). Change in bacterial community structure during in situ biostimulation of subsurface sediment cocontaminated with uranium and nitrate. Appl. Environ. Microbiol. 70, 4911–4920 10.1128/AEM.70.8.4911-4920.2004 15294831 PMC492330

[B52] Palmisano A. Hazen T. (2003). Bioremediation of Metals and Radionuclides: What It Is and How It Works, 2nd Edn Berkeley: Lawrence Berkeley National Laboratory Available at: http://escholarship.org/uc/item/7md2589q

[B53] Payne A. N. DiChristina T. J. (2006). A rapid mutant screening technique for detection of technetium [Tc(VII)] reduction-deficient mutants of *Shewanella oneidensis* MR-1. FEMS Microbiol. Lett. 259, 282–287 10.1111/j.1574-6968.2006.00278.x 16734791

[B54] Prakash O. Gihring T. M. Dalton D. D. Chin K.-J. Green S. J. Akob D. M. Wanger G. Kostka J. E. (2010). *Geobacter daltonii* sp. nov., an Fe(III)- and uranium(VI)-reducing bacterium isolated from a shallow subsurface exposed to mixed heavy metal and hydrocarbon contamination. Int. J. Syst. Evol. Microbiol. 60, 546–553 10.1099/ijs.0.010843-0 19654355

[B55] Rodier J. (1975). L’analyse de l’eau, 5th Edn Paris: Dunod

[B56] Röling W. F. M. van Breukelen B. M. Braster M. Lin B. van Verseveld H. W. (2001). Relationships between microbial community structure and hydrochemistry in a landfill leachate-polluted aquifer. Appl. Environ. Microbiol. 67, 4619–4629 10.1128/AEM.67.5.1995-2003.2001 11571165 PMC93212

[B57] Rooney-Varga J. N. Anderson R. T. Fraga J. L. Ringelberg D. Lovley D. R. (1999). Microbial communities associated with anaerobic benzene degradation in a petroleum-contaminated aquifer. Appl. Environ. Microbiol. 65, 3056–3063 10388703 10.1128/aem.65.7.3056-3063.1999 PMC91456

[B58] Sani R. (2004). Reduction of uranium(VI) under sulfate-reducing conditions in the presence of Fe(III)-(hydr)oxides. Geochim. Cosmochim. Acta 68, 2639–2648 10.1016/j.gca.2004.01.005

[B59] Shelobolina E. Nevin K. Blakeney-Hayward J. Johnsen C. Plaia T. Krader P. Woodard T. Holmes D. Vanpraagh C. Lovley D. (2007). *Geobacter pickeringii* sp. nov., *Geobacter argillaceus* sp. nov. and *Pelosinus fermentans* gen. nov., sp. nov., isolated from subsurface kaolin lenses. Int. J. Syst. Evol. Microbiol. 57, 126–135 10.1099/ijs.0.64221-0 17220454

[B60] Shelobolina E. Vrionis H. Findlay R. Lovley D. (2008). *Geobacter* *uraniireducens* sp. nov., isolated from subsurface sediment undergoing uranium bioremediation. Int. J. Syst. Evol. Microbiol. 58, 1075–1078 10.1099/ijs.0.65377-0 18450691

[B61] Sitte J. Akob D. M. Kaufmann C. Finster K. Banerjee D. Burkhardt E.-M. Kostka J. E. Scheinost A. C. Büchel G. Küsel K. (2010). Microbial links between sulfate reduction and metal retention in uranium- and heavy metal-contaminated soil. Appl. Environ. Microbiol. 76, 3143–3152 10.1128/AEM.00051-10 20363796 PMC2869125

[B62] Snoeyenbos-West O. L. Nevin K. P. Anderson R.T. Lovley D. R. (2000). Enrichment of *Geobacter* species in response to stimulation of Fe(III) reduction in sandy aquifer sediments. Microb. Ecol. 39, 153–167 10.1007/s002480000018 10833228

[B63] Sung Y. Fletcher K. E. Ritalahti K. M. Apkarian R. P. Ramos-Hernández N. Sanford R. A. Mesbah N. M. Löffler F. E. (2006). *Geobacter lovleyi* sp. nov. strain SZ, a novel metal-reducing and tetra chloroethene-dechlorinating bacterium. Appl. Environ. Microbiol. 72, 2775–2782 10.1128/AEM.72.3.1980-1987.2006 16597982 PMC1448980

[B64] Tebo B. M. Obraztsova A. Y. (1998). Sulfate-reducing bacterium grows with Cr(VI), U(VI), Mn(IV), and Fe(III) as electron acceptors. FEMS Microbiol. Lett. 162, 193–198 10.1111/j.1574-6968.1998.tb12998.x

[B65] Van Nostrand J. D. Wu L. Wu W.-M. Huang Z. Gentry T. J. Deng Y. Carley J. Carroll S. He Z. Gu B. Luo J. Criddle C. S. Watson D. B. Jardine P. M. Marsh T. L. Tiedje J. M. Hazen T. C. Zhou J. (2011). Dynamics of microbial community composition and function during in situ bioremediation of a uranium-contaminated aquifer. Appl. Environ. Microbiol. 77, 3860–3869 10.1128/AEM.01981-10 21498771 PMC3127627

[B66] Villanueva L. Haveman S. A. Summers Z. M. Lovley D. R. (2008). Quantification of *Desulfovibrio vulgaris* dissimilatory sulfite reductase gene expression during electron donor- and electron acceptor-limited growth. Appl. Environ. Microbiol. 74, 5850–5853 10.1128/AEM.00399-08 18658285 PMC2547039

[B67] Vishnivetskaya T. A. Brandt C. C. Madden A. S. Drake M. M. Kostka J. E. Akob D. M. Küsel K. Palumbo A. V. (2010). Microbial community changes in response to ethanol or methanol amendments for U(VI) reduction. Appl. Environ. Microbiol. 76, 5728–5735 10.1128/AEM.00308-10 20601514 PMC2935046

[B68] Wagner M. Roger A. J. Flax J. L. Brusseau G. A. Stahl D. A. (1998). Phylogeny of dissimilatory sulfite reductases supports an early origin of sulfate respiration. J. Bacteriol. 180, 2975–2982 9603890 10.1128/jb.180.11.2975-2982.1998 PMC107267

[B69] Wall J. D. Krumholz L. (2006). Uranium reduction. Annu. Rev. Microbiol. 60, 149–166 10.1146/annurev.micro.59.030804.121357 16704344

[B70] Widdel F. Bak F. (1992). “Gram-negative mesophilic sulfate-reducing bacteria,” in The Prokaryotes, eds Balows A. Trüper H. G. Dworkin M. Harder W. Schleifer K.-H. (New York, NY: Springer), 3352–3378

[B71] Wilkins M. Livens F. Vaughan D. Lloyd J. (2006). The impact of Fe(III)-reducing bacteria on uranium mobility. Biogeochemistry 78, 125–150 10.1007/s10533-005-3655-z

[B72] Wu W. Carley J. Gentry T. Ginder-Vogel M. Fienen M. Mehlhorn T. Yan H. Caroll S. Pace M. Nyman J. Luo J. Gentile M. Fields M. Hickey R. Gu B. Watson D. Cirpka O. Zhou J. Fendorf S. Kitanidis P. Jardine P. Criddle C. (2006). Pilot-scale in situ bioremedation of uranium in a highly contaminated aquifer. 2. reduction of U(VI) and geochemical control of U(VI) bioavailability. Environ. Sci. Technol. 40, 3986–3995 10.1021/es051960u 16830572

[B73] Wu W. M. Carley J. Luo J. Ginder-Vogel M. A. Cardenas E. Leigh M. B. Hwang C. Kelly S. D. Ruan C. Wu L. Van Nostrand J. Gentry T. Lowe K. Mehlhorn T. Carroll S. Luo W. Fields M. W. Gu B. Watson D. Kemner K. M. Marsh T. Tiedje J. Zhou J. Fendorf S. Kitanidis P. K. Jardine P. M. Criddle C. S. (2007). In situ bioreduction of uranium(VI) to submicromolar levels and reoxidation by dissolved oxygen. Environ. Sci. Technol. 41, 5716–5723 10.1021/es0623562 17874778

